# The role of sex hormones in the development of Th2 immunity in a gender-biased model of *Trichuris muris* infection

**DOI:** 10.1002/eji.200939589

**Published:** 2009-11-30

**Authors:** Matthew R Hepworth, Matthew J Hardman, Richard K Grencis

**Affiliations:** Faculty of Life Sciences, University of ManchesterManchester, England, United Kingdom

**Keywords:** Cytokines, Comparative Immunology, Parasitic–Helminth, Th1/Th2 cells

## Abstract

*Trichuris muris* infection is an ideal model for defining T-cell-driven immunity, and also provides essential insights that may impact on potential helminth therapies currently in development. Conflicting host variables determine the efficiency of such treatments and we have identified host-derived sex steroid hormones as key factors in the development of immunity. The female-associated hormone 17-β estradiol (E2) significantly enhanced the generation of a Th2 response *in vitro*; however, this stimulatory effect was found to be dispensable for the generation of immunity to *Trichuris* in the gender-biased IL-4KO mouse model. In contrast, the male-associated hormone dihydrotestosterone significantly inhibited the T-cell stimulatory capacity of DC and directly suppressed the immune response of male IL-4KO mice, with worm expulsion restored following castration. This finding was associated with dramatically reduced IL-18 mRNA expression suggesting androgens may act *via* this cytokine to suppress Th2 immunity to *Trichuris*. This study has critical implications for the development and efficacy of potential helminth therapeutics and identifies host gender – specifically sex hormones – as important factors in the development of Th2 immunity in susceptible and immunocompromised mice.

## Introduction

The parasitic helminth *Trichuris muris* has provided key insights into the stimulation and mediation of Th2-associated immunity. Resistance to *Trichuris* is associated with high levels of Th2 cytokines such as IL-4, IL-9 and IL-13 which drive changes in caecum and colon physiology that lead to parasite expulsion 1–3. Studies with helminthic parasites have allowed the elegant delineation of many immunological interactions and mediators that lead to the generation of polarised immune responses, and have themselves recently emerged as intriguing therapeutic candidates due to their ability to modulate and regulate the onset of allergic and inflammatory diseases. Indeed, *T. suis* has shown great promise as a therapy for Crohn's disease 4, 5. In order to successfully interpret the outcome of such studies it is necessary to understand the impact of basic host physiological factors, such as gender and endogenous sex hormone levels, which are seldom addressed in gender-matched inbred animal studies. Gender can play a key role in the outcome of many immunological diseases (reviewed in 6) with females traditionally thought to produce a more vigorous immune response, characterised by higher antibody levels and enhanced adaptive immunity 7, 8. This hyperactive immunity has been suggested to be a contributing factor to the higher incidence in females of autoimmune diseases, such as multiple sclerosis (2:1), rheumatoid arthritis (2:1) and systemic lupus erythrematosus (9:1) than male equivalents 9. Females have also been shown to have higher resistance to many parasitic infections, a finding particularly striking in helminth infections such as *Schistosoma mansoni* and *Trichinella spiralis* 10, 11. Interestingly, gender differences in immune diseases often only present post-puberty, suggesting a causative role for sex steroid hormones in this bias 12.

In order to determine the impact of host gender, and specifically host sex hormones, on the development of immunity to *Trichuris* we utilised a recently described gender-biased model of Th2 immunity (IL-4KO BALB/c) 13. These mice are compromised in their ability to generate an efficient Th2 response, necessary for parasite expulsion, as they lack IL-4 – a key Th2 polarising cytokine. IL-4KO mice on the C57BL/6 genetic background are unable to expel the parasite regardless of host sex. Interestingly, however, we have previously identified a novel gender bias in this knockout when presented on a BALB/c background. Despite high levels of the Th1 cytokine IFN-γ female IL-4KO BALB/c mice mount an unusually delayed Th2 response, associated with T-cell and accessory NK cell-derived IL-13 – and an associated decrease in the levels of the pro-inflammatory cytokines TNF-α and IL-6 (13, 14), which combine to mediate worm expulsion. Conversely, male littermates are unable to expel the parasite and retain high adult worm burdens, a phenotype found to be dependent on IL-18 13.

Although the immunological mechanisms behind this gender difference are now partially characterised the host factors that determine the gender bias are poorly understood. Circulating sex steroid hormones are known to have powerful immunomodulatory capacities. In particular, female sex hormones including 17-β estradiol (E2), the predominant estrogen isoform and progesterone have been extensively studied due to their necessity for foetal tolerance during pregnancy 15. Synthesis of sex hormones takes place mainly in the gonadal tissues of mice (ovaries and testes), although the conversion of the precursor dehydroepiandrosterone into E2 or the most potent androgen dihydrotestosterone (DHT) can also occur *via* enzymatic conversion in peripheral tissues. E2 can act directly on a range of lymphocytes including CD4^+^ T cells, B cells, CD8^+^ T cells, Macrophages and DC 16, 17, which express both estrogen receptor (ER) isoforms; ER-α and ER-β. Moreover, the ER-β isoform is highly expressed in colonic tissue, the host niche for *Trichuris* 18. E2 may then signal through genomic mechanisms (*via* binding to estrogen response elements on promoters) or *via* non-genomic mechanisms (*e.g*. *via* modulation of AP1/SP1 transription factors) to alter the expression of numerous important immunological genes 19, 20. Similarly the androgen receptor is also present on a range of immune cells, including T cells and can alter signalling *via* comparable mechanisms 21.

Previous reports have identified an immunoprotective effect of E2 during *T. spiralis* infection resulting in a reduced muscle larvae burden 22. In addition removal of circulating steroid hormones *via* ovariectomy (OVX) of *Strongyloides venzuelensis* infected females resulted in an increase in adult worm burdens comparable with susceptible males. In contrast castration (CSX) of males infected with the same parasite reduced worm burdens in a complementary manner 23. Finally, pre-treatment of female mice with testosterone prior to *T. gondii* infection reduced intestinal pathology *via* an increase in IFN-γ-mediated parasite killing 24.

Thus, in this study we aimed to test the hypothesis that host sex hormones could directly modulate the immune response to *Trichuris* and thus, account for the gender-biased development of Th2 immunity. *In vitro* studies identified the contrasting roles for major female (E2) and male (DHT) steroid hormones in the tuning of DC during antigen pulsing, and their ability to stimulate a subsequent T-cell response. Furthermore, *via* surgical CSX we demonstrate a key suppressive role for androgens in the suppression of resistance to *Trichuris* in male IL-4KO BALB/c mice.

## Results

### Gender is an important factor in disrupted immunity to *T. muris* infections

**Figure 1:**
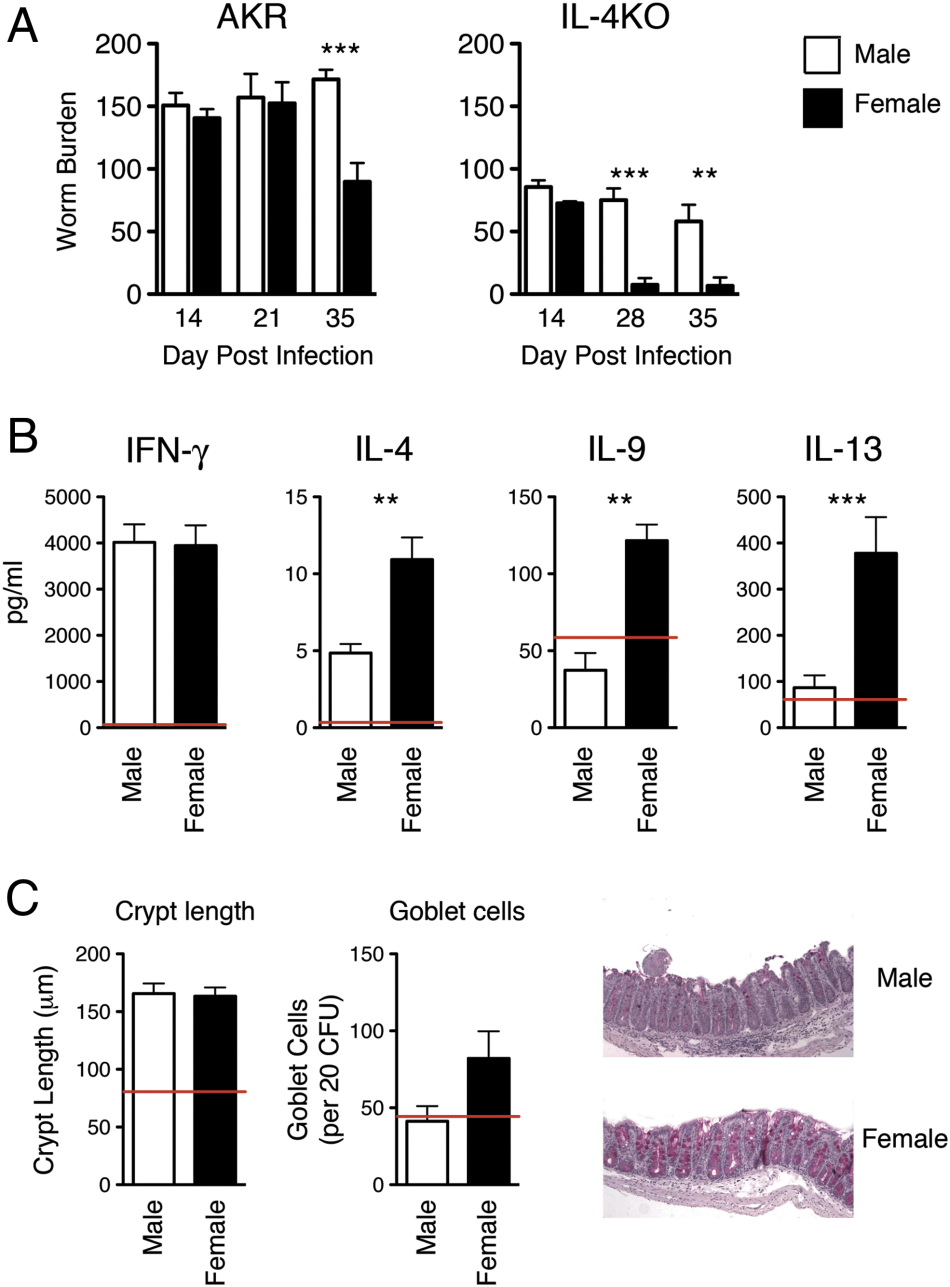
Female mice, but not male litter mates show enhanced Th2-associated immunity in susceptible or slow responder strains. (A) AKR and IL-4KO BALB/c mice were infected with *T. muris* eggs and adult worm burdens assessed at day 14, 21 or 28 and 35 p.i. (B) MLNC from AKR mice were restimulated with *T. muris* E/S antigen after 40 days p.i. and cytokine production assessed *via* CBA. (C) Caecal histology of infected male and female AKR mice was compared at day 40 p.i. Data represent two independent experiments of *n*=10±SEM. ^*^p≤0.05, ^**^p≤0.01, ^***^p≤0.001, Kruskal Wallis ANOVA. Average naïve levels indicated by red lines.

Gender plays a key role in determining resistance or susceptibility in mice with disrupted Th2 immunity in response to the intestinal dwelling nematode *T. muris*. Female IL-4KO BALB/c mice demonstrate a surprising ability to mount a delayed Th2 response and thus, expel *Trichuris* as late as day 28 post-infection (p.i.) (Fig. 1A). This response, a full week later than the typical point of expulsion for immunocompetent animals is mediated by IL-13, recently identified as being both T cell and NK cell derived (13, 14). The AKR mouse strain, considered to be highly susceptible to *Trichuris* infection, develop a chronic infection associated with high adult worm burdens at day 35 p.i. and high levels of IFN-γ and other pro-inflammatory cytokines – at which point infection may only be cleared by anti-helminthic treatment 25. Thus, to investigate whether gender is a common correlate to immune responses in sub-optimal responders we performed a comparison of male and female AKR mice and also observed a surprising gender difference. In a manner similar to IL-4KO BALB/c females the AKR female mice developed a late response to *Trichuris*, which was absent in males, that resulted in a statistically significant reduction (*p*≤0.001) of adult worm burdens in the caecum (Fig. 1A, day 35). In contrast to IL-4KO BALB/c females this reduction did not represent the onset of expulsion as similar worm numbers were found to be present several weeks later (data not shown). In line with the overall chronic nature of AKR infection no differences were observed in the production of the Th1 cytokine IFN-γ in response to *Trichuris* in male *versus* female mice, as measured in MLN cell (MLNC) supernatants *via* cytokine bead array (CBA) (Fig. 1B). Surprisingly, however, female-derived MLNC produced significantly higher levels of IL-4, IL-9 and IL-13 after *T. muris* excretory/secretory proteins (E/S) restimulation at day 40 p.i. than those derived from males (Fig. 1B). Caecal histology was assessed in sections of formalin fixed tissue from these mice and found to directly correlate with cytokine production in the draining LN as both sexes of AKR mice displayed significantly increased crypt lengths compared to naive mice at day 40 p.i. (typically associated with IFN-γ and pro-inflammatory cytokines 25). In contrast, only female mice had enhanced goblet cell numbers in comparison to naive mice at this time, indicative of IL-13-driven goblet cell hyperplasia (Fig. 1C). Thus, these data confirm that in both Th2 disrupted mice and susceptible AKR mice, host gender determines the response to infection.

### E2 enhances the Th2 stimulatory capacity of DC *in vitro*

**Figure 2:**
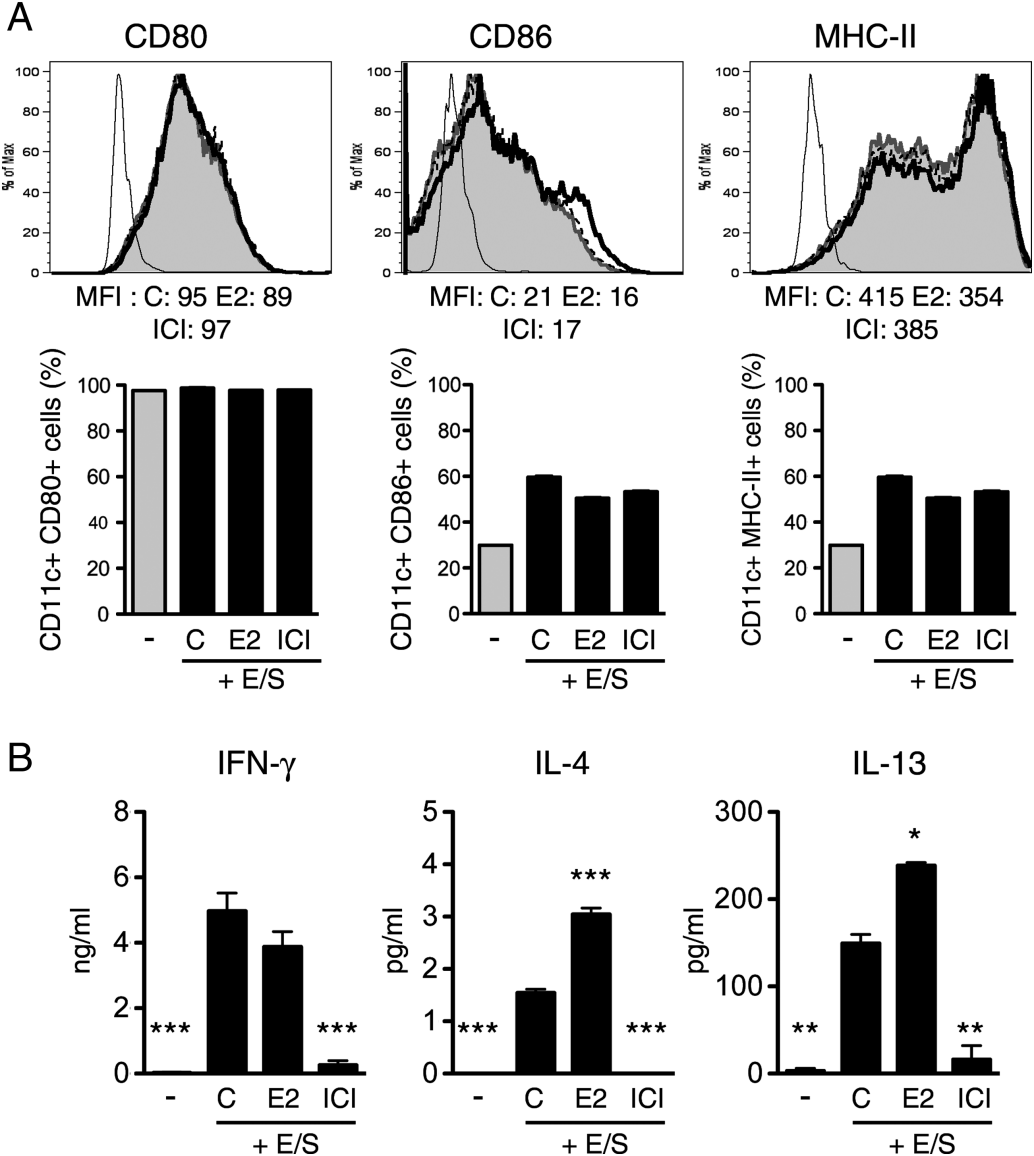
Treatment of BMDC with E2 during *T. muris* E/S pulsing enhances their Th2 priming capacity. (A) BMDC were pulsed with *T. muris* E/S(C, thick black line) and in combination with 10^−10^ M E2 (grey fill) or with E2 and 10^−8^ M ICI 182 780 (ICI, dotted black line). The percent and MFI of CD11c^+^ DC expressing CD80 and CD86 and MHC class II are indicated. Isotype controls are indicated by thin black lines. (B) DC were co-cultured with purified CD4^+^ T cells and following stimulation cytokine production in the co-culture supernatants was assayed *via* CBA. All treatments represent triplicate samples±SEM and the data are representative of three independent experiments. ^*^p≤0.05, ^**^p≤0.01, ^***^p≤0.001, Mann–Whitney *t*-test.

The findings in IL-4KO BALB/c and AKR mice *in vivo* suggest that host gender can play a key role in the development of immunity to *Trichuris*. Sex steroid hormones have been demonstrated to have dramatic effects on the development of immunity in both disease and parasitic infections models 10, 26, 27 and thus, we hypothesised that estrogenic hormones were directly responsible for the observed gender bias in Th2 responses to helminth infection. In order to test this, female WT BALB/c BM-derived DC (BMDC) were pulsed for 24 h with or without *Trichuris* E/S protein alone, or in the presence of 10^−10^ M E2. In addition DC were pulsed in combination with E2 and the ER antagonist ICI 182, 780. An increased number of CD11c^+^ DC expressed CD86 and MHC class II following pulsing with E/S in comparison to unstimulated DC (CD86 – unpulsed 29.9% *versus* E/S C–59.5%, MHC-II – unpulsed 82.1 *versus* E/S C 97.7%), whereas a similar percentage of DC expressed CD80 on their surface (Fig. 2A). Treatment of DC with E2 or in combination with E2 and ICI 182, 780 during pulsing had no significant effects on the percentage or MFI of expression of these classical co-stimulatory markers (Fig. 2A, grey fill and dashed black line, respectively.) DC were then co-cultured with purified CD4^+^ T cells for 3 days, prior to stimulation with IL-2 and a further 3 days of co-culture. T cells co-cultured with E/S pulsed DC produced significantly higher levels of the Th2 cytokines IL-4 and IL-13 in comparison to T cells co-cultured with unpulsed DC (Fig. 2B). Interestingly, DC cultured in E2 containing media during pulsing showed enhanced production of IL-4 and IL-13 in comparison to control cultures (*p*≤0.001 and *p*≤0.05, respectively), whereas treatment of DC did not enhance stimulation of IFN-γ production. Furthermore, inhibition of ER during pulsing completely abrogated cytokine production (*p*≤0.001 for all cytokines *versus* control cultures), confirming previous reports suggesting that E2 signalling is essential for normal DC development and function 28, 29. Thus, E2 can enhance the Th2 stimulating capacity of DC *in vitro*.

### OVX of female IL-4KO BALB/c mice does not prevent expulsion of Trichuris

**Figure 3:**
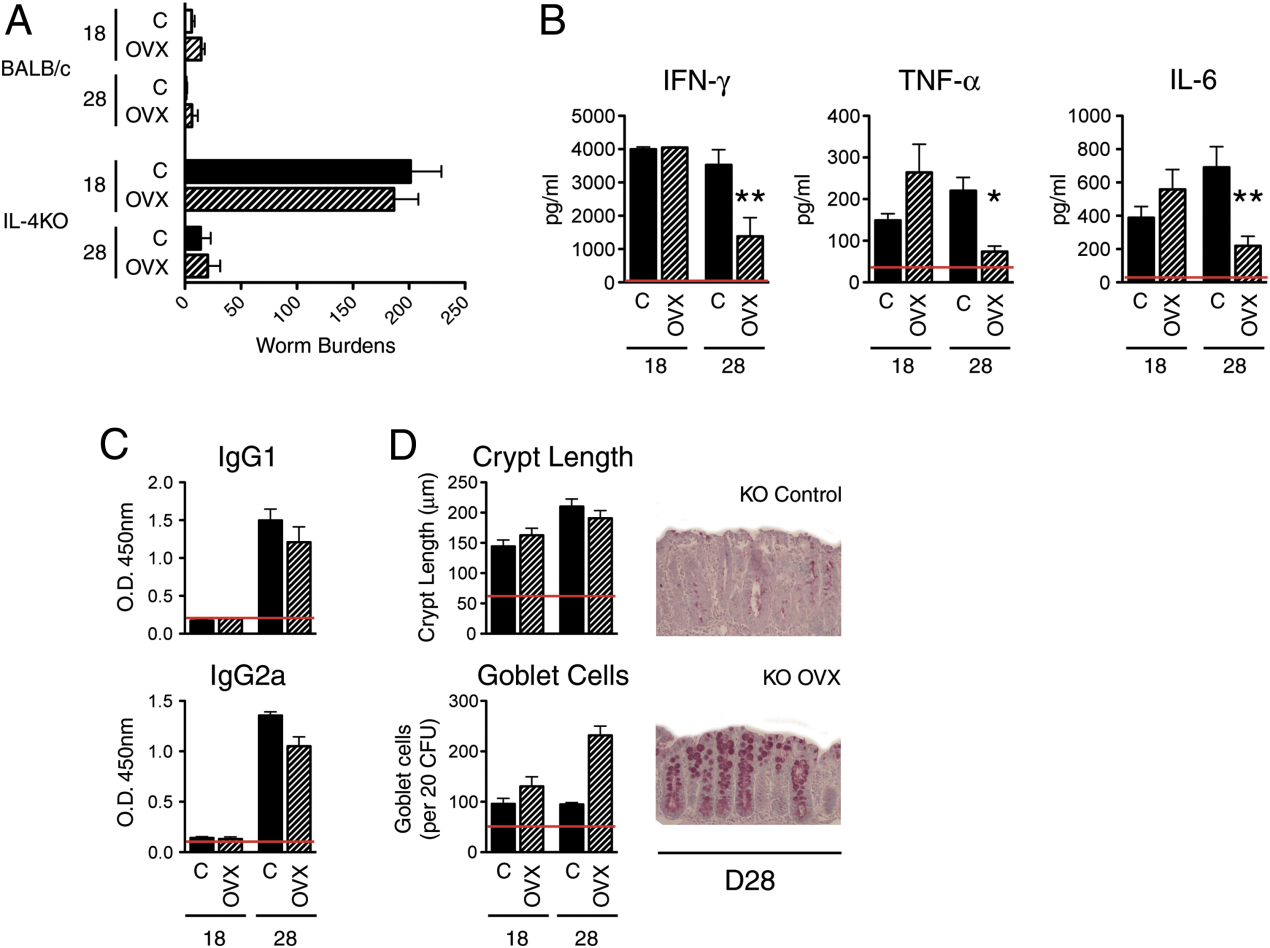
OVX of female IL-4KO mice does not prevent delayed expulsion of *T. muris*. (A) Adult worm burdens in the caecum and proximal colon of intact (C) and OVX WT control and IL-4KO BALB/c mice were assessed at day 18 and 28 p.i. alongside. (B) Levels of pro-inflammatory cytokines from E/S restimulated MLNC. (C) *T. muris*-specific antibody isotype levels in the sera. (D) Caecal histology was assessed in control and OVX female IL-4KO mice. Data represent two independent experiments of *n*=5±SEM. ^*^p≤0.05, ^**^p≤0.01, ^***^p≤0.001, Kruskal–Wallis ANOVA. Average naïve levels indicated by red lines.

In order to determine whether female sex hormones are directly responsible *in vivo* for the late expulsion of *Trichuris* in the gender-biased IL-4KO BALB/c model, female mice were surgically ovariectomised and left for 4 wk in order to achieve negligible baseline circulating levels of estrogenic hormones. Intact and OVX mice were subsequently infected with a high dose of *Trichuris* and worm burdens assessed at day 18 p.i. and day 28 p.i. OVX had no effect on the expulsion of *Trichuris* in these mice as both OVX and intact mice retained similarly high numbers of adult worms at day 18 p.i., and expelled their worm burdens by day 28 p.i. Thus, lack of female sex steroid hormones in these mice did not inhibit expulsion (Fig. 3A). As enhanced immunity in this model is also associated with a decrease in pro-inflammatory cytokines, the levels of TNF-α and IL-6 were assessed in *T. muris* E/S restimulated MLNC culture supernatants during infection. OVX mice displayed an dramatic reduction of these pro-inflammatory cytokines at day 28 p.i., as well as IFN-γ, which is produced at consistently high levels in IL-4KO BALB/c mice in response to *Trichuris* (Fig. 3B). Surprisingly, no Th2 cytokines were detected at this time point, suggesting the transient peak in the production of these cytokines, previously shown to be associated with worm expulsion, may already have passed (data not shown, 13). Both control and OVX mice exhibited goblet cell hyperplasia in comparison to naïve mice at day 28 p.i. (Fig. 3D). However, OVX mice surprisingly demonstrated higher numbers in response to *Trichuris* in comparison to control mice at day 28 p.i. (Fig. 3D), whereas both groups had comparable levels of E/S specific IgG1 and IgG2a in the sera at this time (Fig. 3C). Thus, taken together these finding indicated OVX mice have small changes in their response to *Trichuris* but that worm expulsion remained unaffected.

### DHT inhibits the stimulatory capacity of DC *in vitro*

**Figure 4:**
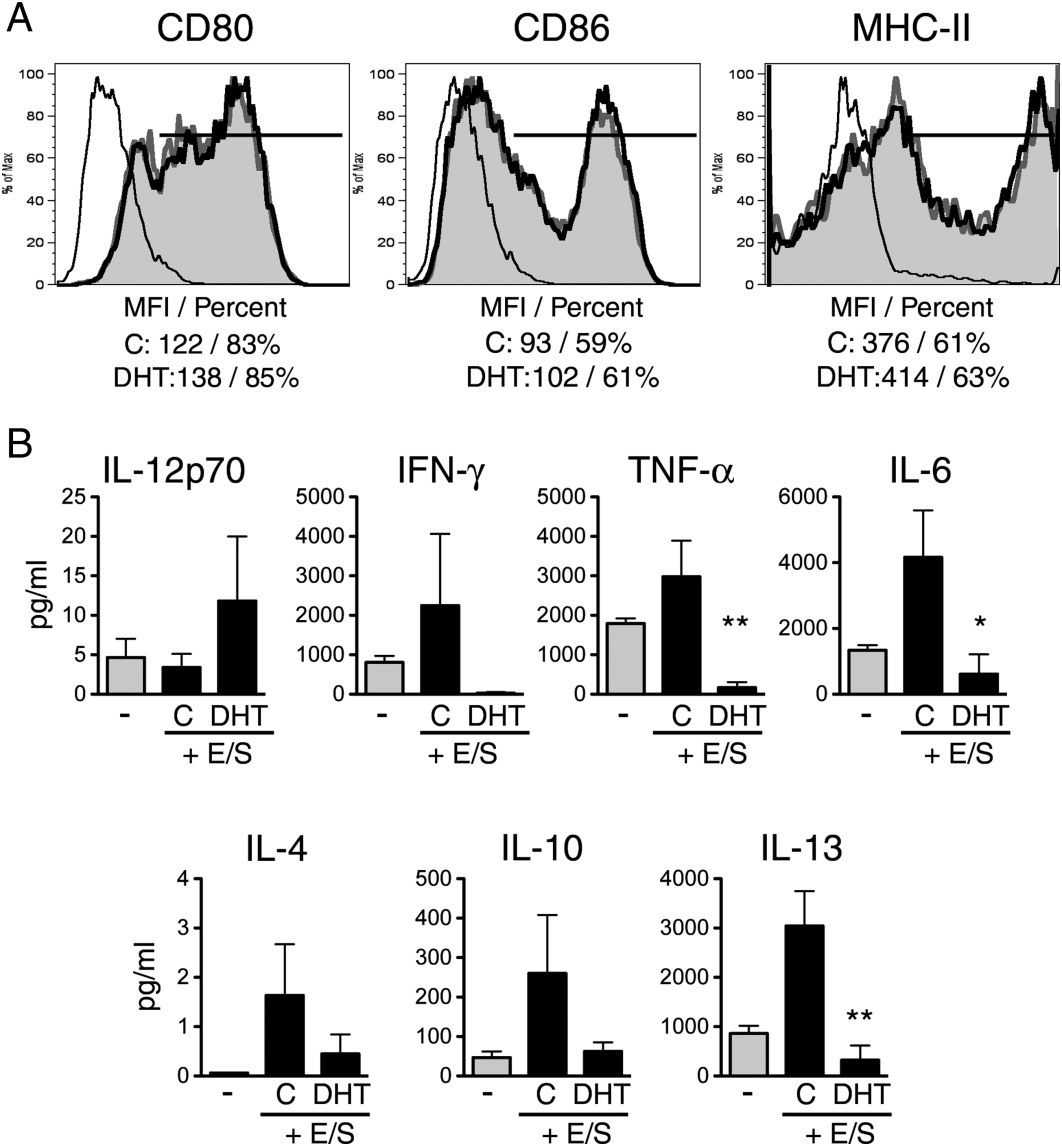
DHT treatment of BMDC inhibits stimulation of T-cell cytokine production. (A) DC were treated with 10^−7^ M DHT during pulsing with *T. muris* E/S and (B) co-cultured with purified CD4^+^ T cells. Cytokine levels in coculture supernatants measured *via* CBA. All treatments represent triplicate samples±SEM and the data is representative of two independent experiments. ^*^p≤0.05, ^**^p≤0.01, ^***^p≤0.001, Mann–Whitney *t*-test.

Although E2 enhanced the Th2 stimulating capacity of DC *in vitro* it was found to be dispensable *in vivo* for the late induction of Th2 immunity in the gender-biased IL-4KO BALB/c mouse model of *Trichuris* infection. Thus, one alternative explanation was that rather than an enhanced resistance to infection in females; male mice were instead compromised in the generation of Th2 immunity, thus, developing a chronic *Trichuris* infection. Androgenic hormones also possess significant immunomodulatory activity, with DHT considered the most potent androgen *in vivo*. Moreover DHT, unlike testosterone, is not subject to conversion into estrogens *via* the enzyme aromatase 30. BMDC from male WT BALB/c were exposed to 10^−7^ M DHT during pulsing with E/S antigen and co-cultured with T cells as previously described. Exposure of DC to DHT during antigen priming had no significant effect on the subsequent upregulation of the co-stimulation markers CD80, CD86 and MHC class II as assessed *via* comparison of the percentage and MFI of expression of CD11c^+^ cells *via* flow cytometry (Fig. 4A). Upon co-culture with E/S pulsed DC, CD4^+^ T cells produced increased amounts of the Th2 cytokines IL-4, IL-10 and IL-13 as compared with those co-cultured with unpulsed DC, with only a mild increase in IFN-γ and TNF-α observed, suggesting an inherent Th2 priming ability of *Trichuris* E/S (Fig. 4B). However, when T cells were co-cultured with DHT-treated E/S pulsed DC there was a dramatic suppression of the Th2 priming ability with a complete ablation of IL-4, IL-10 and IL-13 production (Fig. 4B, bottom panel) (*p*≤0.01 IL-13 DHT treated *versus* control cultures). Furthermore, the production of pro-inflammatory cytokines (IFN-γ, TNF-α and IL-6) was also reduced to levels comparable to, or below that of T cells cultured with unpulsed DC (*p*≤0.01 TNF-α and *p*≤0.05 IL-6 DHT treated *versus* control cultures), although a non-significant trend towards increased levels of the Th1 priming cytokine IL-12p70 was consistently detected in all replicate experiments (Fig. 4B, top panel). Abrogated T-cell cytokine production was unlikely to be a result of cell death as we measured over 90% viability *via* FSC/SSC comparison using a flow cytometer and trypan blue exclusion assay (data not shown). Thus, this data suggests that DHT can inhibit the priming and polarisation of T cells by E/S pulsed DC *in vitro* and prevent the production of Th2 cytokines stimulated by untreated *Trichuris* E/S pulsed DC.

### CSX of male IL-4KO BALB/c mice restores worm expulsion

**Figure 5:**
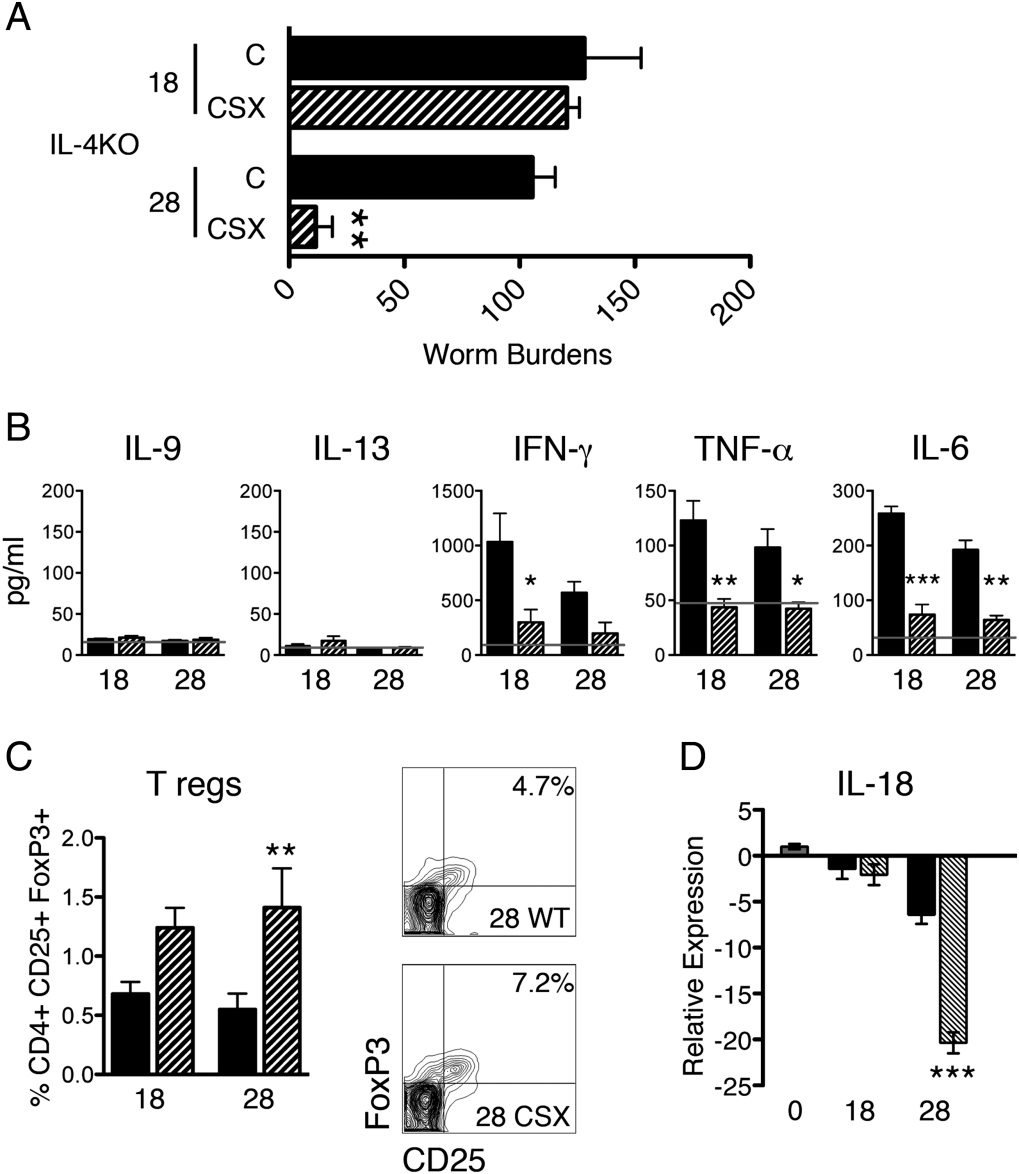
CSX of male IL-4KO mice restores *T. muris* expulsion. (A) Adult worm burdens of intact (C) and CSX male IL-4KO BALB/c mice infected with *T. muris* were assessed at day 18 and 28 p.i. (B) Th2 and pro-inflammatory cytokine production from E/S restimulated MLNC at day 28 p.i. (C) Total number and representative percentages of CD4^+^CD25^+^Foxp3^+^ Treg in the draining LN in CSX and control mice were measured *via* flow cytometry. (D) Relative expression of IL-18 mRNA in the MLN of control and CSX mice assayed during *T. muris* infection. Data represent two independent experiments of *n*=5±SEM. ^*^p≤0.05, ^**^p≤0.01, ^***^p≤0.001, Kruskal–Wallis ANOVA. Average naïve levels indicated by grey line.

*In vitro* studies suggested that androgens might act to inhibit the onset of immune responses to *Trichuris*. However, as E2 was also found to have strong effects *in vitro* which were not essential during infection *in vivo* we sought to address the effects of androgens on the inhibition of immunity to *Trichuris* in the gender-biased IL-4KO BALB/c mouse model. Indeed, surgical CSX of male mice (CSX mice) restored worm expulsion at day 28 p.i. (Fig. 5A). Surprisingly, this expulsion was not associated with a detectable increase in Th2 cytokine production at this time, but did correlate with a marked reduction in IFN-γ, TNF-α and IL-6 as early as day 18 p.i., an observation consistent with expulsion in this model (Fig. 5B). It has recently been shown that Th2 responses to helminth infections are associated with an increase in Foxp3^+^ Treg, which are essential for the regulation of Th2-driven pathology and inflammation during infection 31, 32. CSX mice were found to have increased numbers of CD4^+^CD25^+^Foxp3^+^ Treg in the draining LN in comparison to intact animals (Fig. 5C), thus, suggesting an increase in the regulation of Th2 induced pathology following the removal of androgens.

### Restoration of immunity in CSX male IL-4KO BALB/c correlates with decreased IL-18

We have previously demonstrated a strong inhibitory effect of IL-18 on the development of IL-13 driven worm expulsion in *Trichuris* infection 33. Furthermore, we were recently able to report a direct role for IL-18 in suppressing worm expulsion in male IL-4KO BALB/c mice. Treatment of male IL-4KO mice with anti-IL-18 mAb resulted in restored worm expulsion at day 28 p.i. and significant decreases in IFN-γ, TNF-α and IL-6 in MLN 13. In order to address a potential link between the suppressive effect of androgens and IL-18 in the restoration of expulsion in male IL-4KO mice we assessed the levels of IL-18 mRNA in the draining LN *via* RT-PCR. IL-18mRNA is expressed constitutively at a high level and is processed and secreted following Caspase-1 cleavage 34. CSX mice demonstrated an approximately 20-fold downregulation of IL-18 mRNA expression in comparison to naïve control mice at day 28 p.i. (Fig. 5D). In comparison, intact mice showed significantly reduced downregulation of IL-18 mRNA in response to *Trichuris* (6.4-fold decrease). Thus, this data leads to the intriguing implication that androgens may inhibit expulsion of *Trichuris* in male IL-4KO mice *via* cross-talk with IL-18.

## Discussion

The previously identified gender bias in resistance to *T. muris* in IL-4KO BALB/c mice suggested a complex interplay between the endocrine and immune systems during sub-optimal immune responses 14. Herein, it has been possible to extend the impact of gender to typically “susceptible” mouse strains, such as AKR, which develop chronic infections with *Trichuris*. Furthermore, we show for the first time that gonadal-derived androgens in male IL-4KO mice directly suppress the IL-13-driven expulsion of *Trichuris* worms, and identify a possible link between male sex hormones and IL-18; which also directly suppresses expulsion in the same mice 13. Taken together, these novel findings identify host gender, *via* the direct effect of endogenous sex steroid hormones, as a key immunomodulatory factor that determines the generation of Th2 response to intestinal parasite infections.

*Trichuris* infections have proved to be highly informative with respect to the generation of polarised Th1 and Th2 responses, however, less is known about how the immune system compensates for disruptions in normal priming and T-cell expansion. IL-4KO mice provide an intriguing model to delineate such influencing factors as they lack the classical Th2 cytokine, which mediates important physiological and immunological changes, linked to this response 2, 3. Interestingly, we have previously identified a gender bias in the ability to compensate for a lack of IL-4 as only females were able to expel *Trichuris* infections (in a delayed manner), whereas male counterparts developed persistent infections. In line with this correlation of gender and development of immunity in sub-optimal responders we observed a late decrease in adult parasite numbers in typically “susceptible” female AKR mice, which occurred between day 21 and day 35 p.i. Although this did not represent expulsion of worms, with almost 100 adults maintained on average, the underlying response mirrored that seen in female IL-4KO BALB/c mice at day 26–28 p.i. The response was similarly associated with a late increase in Th2 cytokines and goblet cell hyperplasia, but no reduction in IFN-γ levels. Thus, our findings suggest gender may be a common important host factor in determining immunity in situations where normal Th2 immunity is disrupted.

In order to investigate the basis of such a gender bias in immunity we assessed the impact of host sex hormones on the development of Th2 responses using the previously characterised gender-biased IL-4KO BALB/c model. Sex hormones are known to have dramatic immunomodulatory effects and in line with the predominant literature, we observed a stimulatory role of E2 when applied during *Trichuris* antigen priming of DC. DC pulsed with *Trichuris* E/S preferentially primed T cells to produce Th2 cytokines, suggesting like many other helminth products *Trichuris* E/S has an inherent Th2 polarising ability. Addition of E2 to DC cultures during Ag priming resulted in an enhanced secretion of Th2 cytokines in subsequent T-cell co-cultures (Fig. 2). Although previous studies have determined similar roles for E2 in the generation of both *in vitro* and *in vivo* responses 16, 35, 36, an important novel observation from this study is that the enhancing effects seen during *in vitro* DC priming were not required for the generation of delayed Th2 immunity in female OVX IL-4KO mice *in vivo*. Mice lacking female sex steroid hormones expelled *Trichuris* with comparable kinetics to intact mice and were not disrupted in most key-associated immunological changes. One difference of note was a slight enhancement of goblet cell numbers in OVX mice at day 28 p.i., although control mice also exhibited significant goblet cell hyperplasia at this time (Fig. 3D). This suggests that OVX may have subtle effects on the magnitude of immunity and is likely to reflect a slight delay in the peak of IL-13 production which drives goblet cell differentiation or a failure to efficiently resolve Th2 induced pathological changes in the gut. Thus, taken together this data suggests that although E2 may have the ability to enhance the Th2 priming capacity of E/S pulsed DC, it is not essential for the generation of such a response and worm expulsion *in vivo*.

In stark contrast with these findings we demonstrate a dramatic effect of male sex hormones on the onset of *Trichuris* specific immune responses. *In vitro* treatment of DC with the potent terminal androgen DHT led to a dramatic suppression of E/S DC induced Th2 cytokine production following co-culture with T cells. Thus, as T cells co-cultured with *Trichuris* E/S pulsed DC produce Th2 cytokines under normal control conditions DHT may act to suppress this induction of a Th2 response by APC. Indeed this suppressive effect of DHT on the induction of T-cell immunity was mirrored *in vivo*, as neutralisation of androgens *via* CSX in the IL-4KO mouse model resulted in a restoration of delayed worm expulsion. Although, no significant increase in Th2 cytokines was detected in these experiments, restoration of worm expulsion was associated with marked reductions in pro-inflammatory cytokines (TNF-α, IL-6) and also IFN-γ, as early as day 18 p.i. Moreover, recent data have demonstrated the co-expansion of Treg along with the Th2 response during helminth infections in order to control Th2 cytokine induced pathology 31, 32. In line with this we demonstrate an expansion in the number of regulatory CD4^+^CD25^+^Foxp3^+^ T cells (Treg) in resistant CSX mice. Furthermore, androgen withdrawal has previously been shown to enhance expansion of Treg and naïve T-cell populations, leading to protection from disease in an EAE mouse model 37. Thus, this expansion of Treg in CSX mice is likely to reflect the enhanced requirement for regulated pathology following restoration of the Th2 immune response.

The observed production of high amounts of Th1 and pro-inflammatory cytokines in male mice *in vivo* would appear contradictory to the *in vitro* data, in which these cytokines were also suppressed by DHT. However, it is possible the absence of APC Th2 priming signals *in vivo* may subsequently result in an enhanced pro-inflammatory cytokine production or that this alternate priming is possibly due to bystander stimulation by exposure to bacterial LPS during parasite mediated intestinal damage.

We have previously reported a key link between IL-18 and suppression of worm expulsion in male IL-4KO BALB/c mice 13. IL-18 is an IFN-γ inducing cytokine which has pleiotropic effects including the suppression of Th2 immunity to *Trichuris* and *T. spiralis* 33, 38. IL-18 mRNA is constitutively expressed in many cells in intact mice. Interestingly, CSX mice had a large reduction in IL-18 mRNA in MLN in comparison with intact mice at day 28 p.i., coinciding with the expulsion of worms in CSX mice, thus, one possibility is that the expression of the IL-18 mRNA pool is directly influenced by androgenic hormones *in vivo*. In line with this hypothesis male mice have been shown to produce higher levels of IL-18, which increases mortality, during sepsis in comparison to females 39.

The present data provide important new insights into the role of sex hormones in modulation of Th2 immunity. Furthermore, these findings are an important pre-requisite for the testing of proposed helminth therapy for inflammatory diseases. Hormone levels often directly correlate with many target diseases – for example, decreased androgen levels in males have been shown to correlate with increased inflammatory bowel disease (IBD) in men 40, whereas, post-menopausal women may also differ in their responses to such proposed therapy; hormone replacement therapy (HRT) patients often have reduced symptoms of ulcerative colitis and Crohn's disease – two current target diseases for *T. suis* therapy – compared with non-HRT-treated women 41. Thus, this study provides important insight into sub-optimal Th2 responses to intestinal parasites which may prove useful in the future development of helminth therapies.

## Materials and methods

### Mice and *T. muris* maintenance

IL-4KO BALB/c mice were originally obtained from Dr. N. Noben-Trauth (George Washington University, USA) alongside WT BALB/c control mice and bred under specific pathogen-free conditions at the University of Manchester, UK. AKR mice and additional BALB/c mice used for *in vitro* studies were purchased from Harlan-Olac (Bicester, UK). The intestinal dwelling nematode *T. muris* was maintained in immunodeficient SCID mice and infective embryonated eggs isolated as described previously 42. Mice were infected with 150–200 *T. muris* eggs between the ages of 6 and 8 wk *via* oral gavage and each experimental group contained 5 mice, or 10 mice where indicated. Adult worm counts were assessed *via* longitudinal dissection of infected caeca and proximal colons at the time points indicated. All experiments were performed under the regulations of the Home Office Scientific Procedures Act (1986).

### Surgery

Ovaries (OVX) and Testes (CSX) of IL-4KO BALB/c mice were removed under anaesthesia with a mixture of isoflourane and nitrous oxide with pure oxygen using an established method 43, 44. Briefly, the ventral side of the mouse was shaved, cleaned and the ovaries or testes removed *via* ventral laparotomy. The body wall was subsequently closed using absorbable polyglycolic acid suture (Dexon II, Johnson & Johnson) and the skin sutured with a silk braided non-absorbable suture (Ethicon Mersilk, Johnson & Johnson). Briefly, 0.1 mg/kg buprenorphine analgesia was administered to mice i.p. Mice were left for 4 wk prior to subsequent experimentation in order to allow circulating hormones to reach basal levels.

### Lymphocyte restimulation and cytokine analysis

Single-cell suspensions were prepared from the MLN of infected and naïve mice and plated at 5×10^6^ cells/mL in RPMI 1640 supplemented with 10% FBS, 2 mM l-glutamine, 100 U/mL penicillin and 100 μg/mL Streptomycin (Invitrogen). Cultures were subsequently re-stimulated with 50 μg of *T. muris* E/S for 24 h at 37°C/5% CO_2_. *T. muris* E/S proteins were routinely tested for endotoxin contamination in the lab and found on average to contain negligible levels below the level of test sensitivity. The resulting supernatants were assayed for the levels of IFN-γ, IL-4, IL-6, IL-9, IL-10, IL-13 and TNF-α *via* CBA kit (BD Biosciences) following the manufacturer's instructions and assessed using a BD FACSCalibur and FCAP array software (BD Biosciences).

### BMDC culture and T-cell costimulation assays

BMDC were generated as described previously 45. Briefly, the femurs and tibias of male or female WT BALB/c were isolated and the BM extracted and lysed to remove erythrocytes. Isolated cells were cultured in complete culture media, as detailed above, supplemented with 0.5 mL GM-CSF containing supernatant derived from the Ag8 hybridoma cell line. GM-CSF containing supernatant was replaced every 3 days and after 7 days non-adherent DC collected and assessed for expression of CD11c (BD Biosciences) *via* flow cytometry. In order to assess hormonal modulation of DC priming by *T. muris* E/S cells gender-matched DC were seeded at 5×10^6^ cells/well in a 48-well plate and pulsed with 50 μg *T. muris* E/S or in combination with either 10^−7^ M DHT (male BMDC) or 10^−10^ M E2 (female BMDC (Sigma-Aldrich). Alternatively, DC were pulsed with 500 ng/mL LPS as a positive control. Following pulsing CD11c^+^ DC were assessed for their expression of MHC class II, CD80 and CD86 with fluorochrome conjugated antibodies (BD Biosciences) *via* flow cytometry. In some experiments 0.5×10^6^ pulsed and treated DC were cultured, following thorough washing, with 2.5×10^6^ CD4^+^ T cells purified from naïve WT BALB/c females *via* AutoMacs bead separation (Miltenyi Biotec). After 48 h co-culture cells were supplemented with 20 U/mL rIL-2 (Peprotech) and cultured for a further 72 h prior to quantification of cytokines in culture supernatants.

### Antibody analysis

Sera were isolated from mice *via* cardiac puncture and centrifugation at 15 000×*g* for 15 min. To assess parasite-specific IgG1 and IgG2a, Immunlon IV plates (Thermo Life Sciences) were coated at 4°C overnight with 50 μg/mL of *T. muris* E/S in carbonate/bicarbonate buffer, pH 9.6. Non-specific binding was blocked with 3% BSA in 0.05% Tween 20 PBS prior to addition of sera at a 1:64 dilution. Parasite-specific antibody isotypes were detected using biotinylated rat anti-mouse IgG1 (Serotec) or biotinylated rat anti-mouse IgG2a (BD Biosciences) prior to developing with tetramethylbenzidine (TMB). Relative levels were measured at OD at 450 nm, with reference to 570 nm.

### Histological analysis

Caecal snips were removed from naïve and infected mice and stored in 4% neutral buffered formalin prior to processing and embedding in paraffin wax. Sections of 4 μm were adhered to slides and dewaxed using citroclear and dehydrated prior to staining with periodic acid and Schiff's reagent to visualise goblet cells. Sections were mounted and crypt lengths assessed using Image J software (NIH). The number of goblet cells *per* 20 crypt-forming units was also assessed.

### RNA extraction and real-time PCR

Total MLN RNA was extracted from naïve and infected animals and stored in TRIzol reagent prior to Chloroform and Isopropanol precipitation and extraction. cDNA was subsequently generated *via* reverse transcription using ImProm-II RT (Promega). The relative expression of hypoxanthine phosphoribosyl transferase (HPRT) (Sense 5′ GCGTCGTGATTAGTGATGATGAAC 3′/Antisense 5′ GAGCAAGTCTTTTCAGTCCTGTCCA 3′) and IL-18 (Sense 5′ acagtgaagtaagaggactggctg 3′/Antisense 5′ CAGGTGCATCCATTTCCTCAAAGG 3′) were assessed *via* RT-PCR using a reaction containing SYBR green master mix (Finnzymes), 0.1 μg cDNA and 50 pmol of primers. All reactions were performed with the following cycle conditions: 40 cycles of 94°C 10 s, 60°C 20 s and 72°C for 15 s. Relative expression was determined and standardised to the house-keeping gene and naïve control samples *via* the comparative 2^−ΔΔC(t)^ method. All samples were checked for specificity of amplification *via* Melting curve analysis.

### Statistical analysis

Statistical significance for the data presented, representative of three independent experiments unless indicated, was calculated using a Kruskal Wallis ANOVA followed by a Dunn's *post hoc* test, or *via* Mann–Whitney *t*-test where appropriate. Values of *p*=<0.05 were considered significant.

## Abbreviations

BMDC: BM-derived DC
CBA: cytokine bead array
CSX: castration
DHT: dihydrotestosterone
E2: 17-b estradiol
ER: estrogen receptor
E/S: Trichuris muris excretory/secretory proteins
MLNC: MLN cell
OVX: ovariectomy
p.i.: post-infection
